# Targeted therapy of RET fusion-positive non-small cell lung cancer

**DOI:** 10.3389/fonc.2022.1033484

**Published:** 2022-12-13

**Authors:** Zixiong Shen, Binxu Qiu, Lin Li, Bo Yang, Guanghu Li

**Affiliations:** ^1^ Department of Thoracic Surgery, The First Hospital of Jilin University, Changchun, China; ^2^ Department of Gastrointestinal Surgery, The First Hospital of Jilin University, Changchun, China

**Keywords:** non-small cell lung cancer (NSCLC), RET gene, RET fusion, targeted therapy, cancer biology

## Abstract

Lung cancer has very high morbidity and mortality worldwide, and the prognosis is not optimistic. Previous treatments for non-small cell lung cancer (NSCLC) have limited efficacy, and targeted drugs for some gene mutations have been used in NSCLC with considerable efficacy. The RET proto-oncogene is located on the long arm of chromosome 10 with a length of 60,000 bp, and the expression of RET gene affects cell survival, proliferation, growth and differentiation. This review will describe the basic characteristics and common fusion methods of RET genes; analyze the advantages and disadvantages of different RET fusion detection methods; summarize and discuss the recent application of non-selective and selective RET fusion-positive inhibitors, such as Vandetanib, Selpercatinib, Pralsetinib and Alectinib; discuss the mechanism and coping strategies of resistance to RET fusion-positive inhibitors.

## Introduction

Lung cancer has very high morbidity and mortality worldwide, and the prognosis is not optimistic, and the prognosis is not optimistic. According to a study (1), lung cancer accounted for 11.6% of cancer incidence and 18% of deaths, respectively, in 2020 and likely resulted in over 1.8 million deaths. When lung cancer is contained at the initial site, the five-year survival rate is 56.3% and reaches 29.7% when regional lymph node metastasis occurs, and less than 13% when distant metastasis occurs (2, 3). This dismal prognosis suggests that the efficacy of treatment for lung cancer remains uncertain. Men are 1.89 times more likely than women to develop lung cancer, and 85% of lung cancers are NSCLC (3). Early-stage NSCLC is mainly treated with surgery and chemotherapy. Chemotherapy drugs represented by carboplatin, cisplatin, etoposide, irinotecan, Docetaxel and pemetrexed (4) have limited efficacy, only about 20-30% (5) of NSCLC respond, and most will eventually relapse. As a result, novel techniques for improving patient outcomes are required. Identifying driver genes in lung cancer patients can alter the therapy landscape for lung cancer. Numerous molecular abnormalities have been revealed in NSCLC, and several targeted therapies to treat these abnormalities have also entered clinical trials. These targeted medications appear to have greater efficacy and safety when compared to chemotherapy (6–8), and these therapies are critical for improving lung cancer outcomes, delaying the course of lung cancer, and perhaps regulating disease progression. ALK, ROS-1, NTRK, EGFR, KRAS, BRAF, and RET are the most mutated genes currently being researched (9). Although the incidence of RET gene rearrangement in non-small cell lung cancer is 1% to 2% (10), it is still of great significance to study RET fusion-positive NSCLC due to the high incidence of lung cancer. Tyrosine kinase inhibitors (TKIs) that inhibit RET fusion genes have made some breakthroughs in the past few years, especially Selpercatinib and pralsetinib have been approved by the FDA (11, 12). In this review, we will describe the basic characteristics and common fusion methods of RET genes; analyze the advantages and disadvantages of different RET fusion detection methods; summarize and discuss the recent application of non-selective and selective RET fusion-positive inhibitors; discuss the mechanism and coping strategies of resistance to RET fusion-positive inhibitors.

## RET fusion and its detection

### RET gene

**Figure 1:**
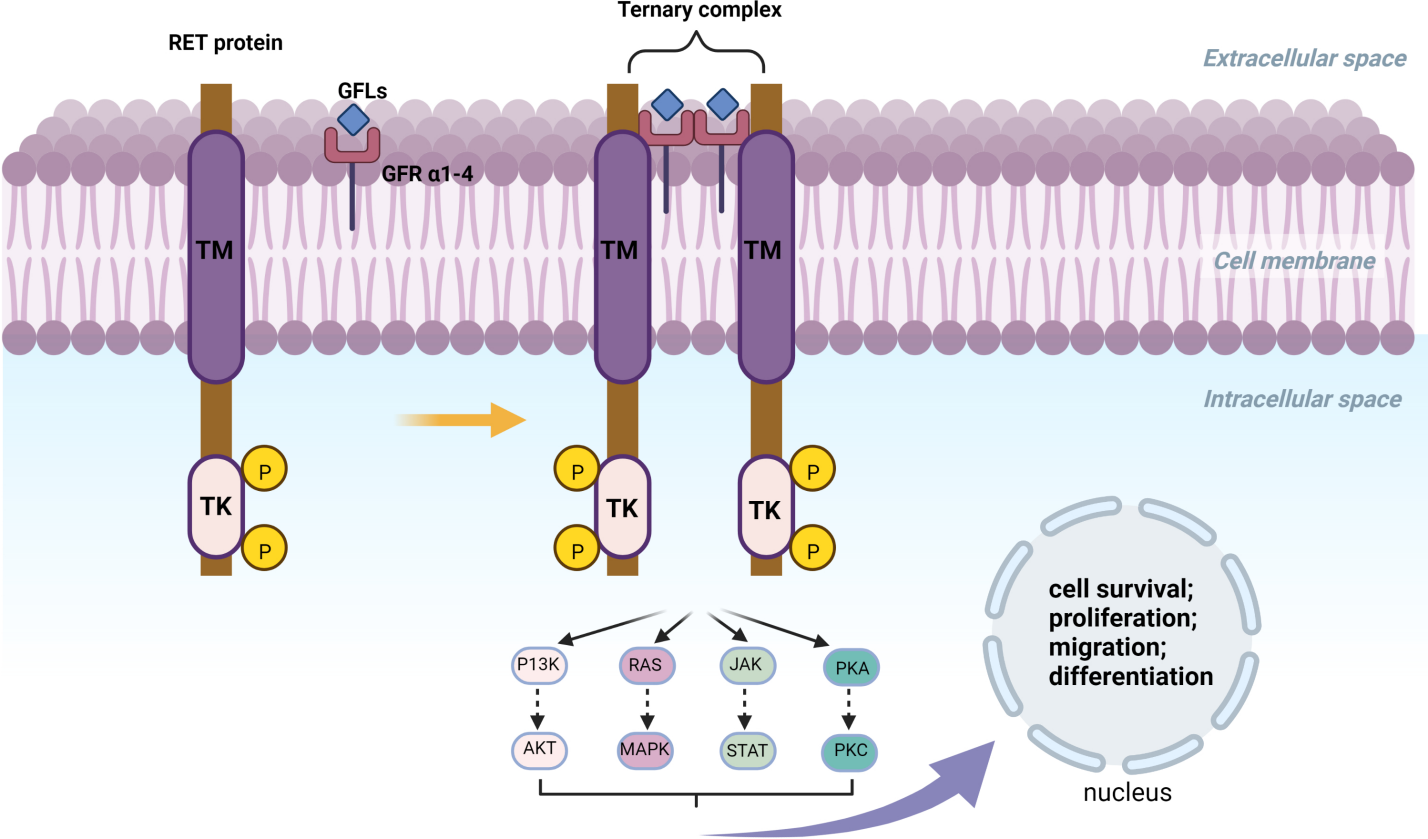
TM, Hydrophobic Transmembrane domain; TK, Tyrosine Kinase domian; KM, Kinesin motor; aa, amino acid. The RET gene forms a ternary complex with GFLs and GFR α1-4. RET fusion can form Ligan-Independent Homodimerzation and further activate or inhibit P13K/AKT, RAS/MAPK, JAK/STAT and PKA/PKC. Activation or inhibition of these pathways is closely related to cell survival, proliferation, migration and differentiation.

The RET proto-oncogene is located on the long arm of chromosome 10 with a length of 60,000 bp, with 21 exons (13). In 1985, Takahashi et al. found a RET fusion gene activated by DNA rearrangements during the transfection of NIH3T3 cells using human T-cell lymphoma DNA (14, 15). RET proto-oncogene located on Chr 10 long arm is a fusion gene that encodes a tyrosine kinase receptor protein with 1076, 1106, or 1114 amino acids which are produced by alternative splicing in its 3 prime regions. RET protein has a tyrosine kinase intracellular domain linked to an outer cysteine-rich extracellular domain and four cadherin-like domains through transmembrane (16, 17). The RET gene forms a ternary complex with Glial cell line-derived neurotrophic factor (GDNF) family ligands (GFLs) and GDNF family co-receptors (GFR α1-4) leading to autophosphorylation of RET-intracellular domain, which activates downstream signaling pathways like PI3K/AKT, RAS/MAPK, JAK/STAT, and PKA/PKC, whose activation or inhibition has an important impact in cell survival, proliferation, migration, and differentiation (18, 19). **Figure 1** illustrates the constitutive patterns of the RET proteins.

RET gene promotes carcinogenesis primarily by gene fusion, point mutation, and amplification, associated with numerous cancers (19, 20). RET gene is involved in the development of embryonic urogenital and neural tissues, involved in the stability of brain tissue, hematopoietic tissue, and urogenital system (20). Point mutations in RET gene have been identified in familial medullary thyroid cancer, multiple endocrine neoplasia type 2 syndrome, pheochromocytoma, and chronic myeloid leukemia (17, 21). Point mutations, RET fusions, and amplification of RET gene are observed in various cancers. RET fusions which occur due to chromosomal rearrangements resulting in RET protein’s C-terminus splicing with the N-terminus of another protein and are seen in papillary thyroid carcinomas, NSCLC in young never-smokers, advanced disease, and poorly differentiated populations (9, 22, 23). RET fusions occur in a variety of ways, the most common being KIF5B-RET and CCDC6-5B. The data from 12 countries and 29 centres shows that KIF5B-RET (62.4%) and CCDC6-5B (20.8%) were found in 173 lung cancer patients with a positive RET fusion profile (24). And they found that stage IV RET fusion-positive lung cancer seems to have a higher brain metastasis rate of 25% (33/133). A study (25) from China used DNA next generation sequencing (NGS) to profile RET fusions in 12,888 lung cancer patients. In this study, RET fusions occurred in 1.1% of cases, with KIF5B-RET (62%) and CCDC6-5B (21%) being the two most common fusions. They also found some fusion modes that had not been found before, such as DNER, DPP6, FGD5. Another study (26) included 9,471 patients with NSCLC, and detected 167 (1.7%) patients with a RET fusion using DNA NGS. The most common fusion partner was KIF5B (68.2%, 114/167), followed by CCDC6 (16.8%, 28/167). Notably, they found that while in EGFR/KRAS/BRAF/ALK-negative NSCLC patients, the prevalence of RET rearrangement was 8.79% (29/330). This is similar to some previous reports (10), indicating that RET and other fusions are possibly exclusive. **Table 1** lists the variation of the RET genes as reported in the literature.

**Table 1:** Common RET gene fusion patterns.

	KIF5B	CCDC6	ERC1	ERCC6	NCOA4	TRIM33	EML4	FBXL7	GRIPAP1	KIF13A	TIMM23B	Others^1^	Total
Drilon et al., 2018 (24),	108	36		3	2		4	2	2	2	2	12	173
Shi et al., 2022 (25),	85	29	3		2	2				1		15	135
Feng et al., 2022 (26),	114	28			2							23	167

^1^Some other fusion patterns where only 1 case was detected, such as DNER, DPP6, FGD5, GADL1, GLI3, GPRC6A, IL1RAPL2, KIAA1598, MALRD1, PRKAR1A, SPECC1, TLN1, ZNF33B, TRIM24, EPHA5, MYO5C, EML4, RUFY3, KIAA1468.

**Figure 2:**
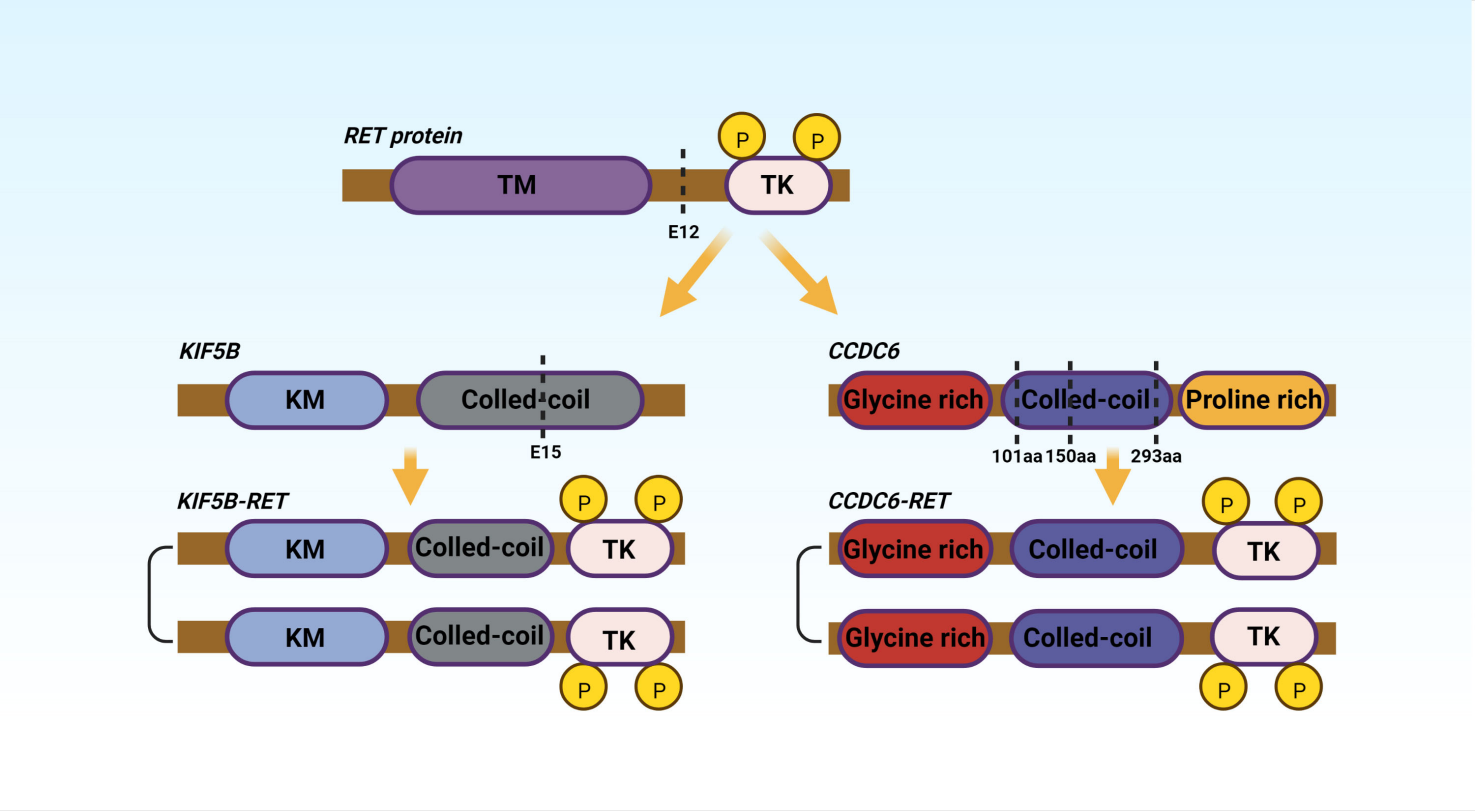
TM, Hydrophobic Transmembrane domain; TK, Tyrosine Kinase domian; KM, Kinesin motor; aa, amino acid. The figure shows the two most common fusions, KIF5B-RET and CCDC6-RET.

Researchers (27) discovered that RET-KIF5B fusion-positive was primarily localized in exons 12 of RET protein and exons 15 of KIF5B in 371 NSCLC patients. CCDC6 dissociates at its amino acids 101, 150, and 293, and then forms a fusion protein with the RET gene (28). **Figure 2** depicts typical rearrangement patterns of RET fusion proteins.

### The detection of RET fusions

There are many detection technologies for RET fusion, and each method has its advantages and disadvantages and usage scenarios. Test results are affected by factors such as the type and quantity of genetic variants detected, specimen type, specimen quantity and quality, and laboratory conditions. The most commonly used methods for detecting RET fusions are Immunohistochemistry (IHC), fluorescence *in situ* hybridization (FISH), next-generation sequencing (NGS), reverse transcription-polymerase chain reaction (RT-PCR), and PCR/Sanger. IHC detects protein, which can identify known and unknown locations which are not only inexpensive but quick as well. The IHC detection platform is highly accessible, cheap and fast, but the availability of antibodies is poor, and there are certain false negatives and false positives (both up to 40%) (29). IHC mainly uses the principle of antigen-antibody specific binding, and determines the antigen (polypeptide and protein) in tissue cells by chemical reaction to develop the color of the labeled antibody, and performs localization, qualitative and relative quantitative detection technology. IHC has been widely used to detect fusion genes such as ALK. However, when used to detect RET fusions, its sensitivity and specificity are unreliable, with studies reporting sensitivities of 50%-100% and specificities of 30%-90% (30, 31), and thus may only be suitable for extensive primary screening check, but not for a standalone test. In addition, IHC cannot detect partner genes (22), so the widespread use of IHC in RET detection is not recommended.

FISH works by labeling DNA or RNA probes with specific nucleotide molecules that can find, qualitatively, and quantitatively assess DNA or RNA sequence at the probe’s site (22, 32). FISH has the highest sensitivity for the detection of classical RET fusions (KIF5B-RET, CCDC6-RET) at 86%-91.7% and 95%, respectively, while other non-classical fusions such as NCOA4-RET have a sensitivity of only 66.7% (33, 34). The FISH specificity has been reported to reach 50.6%-99% (33, 34). But FISH is associated with demerits like high detection cost, extended detection time, need for professional supervision, and high subjectivity (35). FISH is the “gold standard” for detecting gene translocations/fusions but has false negatives for rare variants, and lacks cut-off values (36).

RT-PCR performed well in terms of specificity(77%-100%), sensitivity(91.43%-99%) and automation (37–40). However, sensitivity may be reduced when there are several fusion partners, and RT-PCR can only detect known sites, not new or unknown ones (10, 31). The detection material of RT-PCR is known fusion mRNA, but cannot detect rare fusion partners not covered by primers (41). Given the potential for false positives with FISH, FISH is often used as primary screening in a broad range of multikinase inhibitor (MKI) screening studies and in phase II clinical trials, and further validated with RT-PCR or RNA NGS (23, 42), especially in the face of For atypical FISH results (such as single color, signal amplification, etc.).

NGS is a modernization of classic Sanger sequencing, including DNA NGS and RNA NGS, with the benefits of high throughput, ease of use, high accuracy, and large-scale gene screening (31). Therefore, NGS not only saves specimens, but also saves time waiting for test results. For targets with lower mutation frequencies, the advantages of NGS are more obvious. DNA NGS can be used to detect several mutations simultaneously, the reported sensitivity ranges from 87.2% to 100%, and the specificity ranges from 98.1% to 100% (43–46). However, its sensitivity is lower than that of RNA NGS due to its limited coverage of intronic areas and is associated with the problem of false positivity. Furthermore, the rearrangements found by DNA NGS may not produce fusion. Unlike DNA NGS, RNA NGS does not have the disadvantage of intron coverage and may obtain fusion partner information simultaneously, allowing it to detect gene expression directly. However, RNA NGS detection is generally limited to specific common fusion types causing rare fusions to be missed, and the sensitivity of RNA NGS is probably affected by the design of the detection product (47). The sensitivity and specificity reported in the literature can reach 88.46%-100% and 95.83%-100% (48, 49). Other detection technologies such as NanoString technology/PCR/Sanger sequencing have a high cost, unpopular detection instruments, and time-consuming. Even with high sensitivity and specificity, they are not suitable for routine clinical applications (50, 51). For the detection of RET fusion-positive, Yang et al. advocate a combination of screening and confirmatory assays (33). They make the following recommendations: DNA NGS is recommended as the primary screening tool due to its broad sensitivity to all RET fusions; atypical RET mutations with novel fusion partners, antisense fusions, or intergenic regions should be sequenced by RNA NGS for further evaluation; when DNA NGS is not available, use FISH for screening, and use RNA NGS to confirm atypical positive and borderline negative FISH results. **Table 2** compares several commonly used detection technologies.

**Table 2:** Comparison of several detection technologies.

Technologies	IHC	FISH	RT-PCR	DNA NGS	RNA NGS
specificity	+++	++++	+++++	+++++	+++++
sensitivity	+++	++++	+++++^b^	+++++	+++++^c^
charge	++	+++++	+++	++++	++++
materials	protein	DNA/RNA	RNA	DNA	RNA
detection of partner	no	no/yes^a^	yes/no^b^	yes/no^b^	yes/no^b^
time consumption (d)	1~2	2~3	2~3	5~7	5~7

IHC, immunohistochemistry; FISH, fluorescence in situ hybridization; RT-PCR, reverse transcription polymerase chain reaction; NGS, generation sequencing. a. When using probes with specific fusion partners; b. Unable to detect rare fusion partners not covered by primers; c. Related to product design.

## The relationship between RET fusions and NSCLC

The incidence of RET fusion-positive NSCLC is similar in men and women. RET fusion-positive NSCLC has typical clinical features, such as younger age at onset and a low smoking rate (more than 60% of patients have never smoked) (52). Adenocarcinoma occurs in 98% of RET fusion-positive NSCLC patients, and 70% of RET fusion-positive NSCLC patients are in stage IV at diagnosis (43, 52). This implies that NSCLC patients with RET gene rearrangement have a poor prognosis and are more likely to develop distant metastasis. However, it does not rule out the possibility that non-metastatic patients are rarely screened.

RET gene positivity in non-small cell lung adenocarcinoma is approximately 1% to 2%, RET fusion mutations and some other genetic mutations (eg, EGFR receptor mutations, ALK gene rearrangements) are mutually exclusive (42). The frequency of RET rearrangements increases in the absence of other oncogenic driver mutations, and the prevalence of RET rearrangements at this time is estimated to be approximately 5% (42). RET fusion-positive NSCLC patients show poorer tumor cell differentiation, more signet ring cell subtypes, and smaller primary lesions (<3 cm) when compared to ALK rearrangement, EGFR receptor mutation, and ROS-1 fusion-positive patients (22, 51, 53).

An in-frame fusion of the kinesin family 5B gene (KIF5B) with the RET gene was the first RET fusion discovered in NSCLC (54). The oncoprotein is induced by RET gene and its fusion partner gene, which also causes the activation of associated signaling pathways, which can make cells malignant and progress toward lung cancer. The positive RET fusion gives patients without harmful traditional targeted genes fresh hope, and it may become a successful target for NSCLC patients’ targeted therapy in the future.

## RET fusion-positive NSCLC inhibitors

Researchers conducted multiple clinical trials of Vandetanib, Cabozantinib, and Lenvatinib in RET fusion-positive thyroid cancer. These drugs have even been approved to treat thyroid cancer (15). Several multi-kinase inhibitors (MKIs) targeting RET fusion-positive NSCLC have also been studied clinically or pre-clinically.

Numerous studies (55–57) have indicated that MKI might induce more noticeable adverse effects such as nausea, diarrhea, rash, and elevated blood pressure, leading to dose decrease or drug discontinuation. Due to the low pharmacokinetics and non-selectivity of MKI, its treatment of RET fusion-positive NSCLC is limited, especially in terms of disease remission rate and disease progression control. Some selective TKIs have achieved breakthroughs in clinical trials in the past two years, and some drugs have also been approved by the EMA or the FDA. The representative drugs Pralsetinib and Selpercatinib have shown good efficacy and safety. The FDA previously granted selpercatinib (LOXO-292) accelerated approval in 2020 for RET fusion–positive metastatic NSCLC, excitingly, Selpercatinib was authorized by the FDA as first-line regular therapy for RET fusion-positive advanced or metastatic NSCLC in Nov 2022 (11). The FDA also granted Pralsetinib (BLU-667) accelerated approval in Sep 2020 (12). Selpercatinib was approved as second-line therapy for RET fusion-positive NSCLC by Swissmedic and the EMA in 2021 (58). We will summarize the recent application of non-selective and selective RET fusion-positive inhibitors, and discuss the mechanism and coping strategies of resistance to RET fusion-positive inhibitors.

### Non-selective RET fusion-positive inhibitors

Vandetanib is an oral multi-target inhibitor with anti-angiogenic and anti-RET properties that target RET, VEGFR, and EGFR signaling pathways (59). FDA approved vandetanib, which primarily inhibits the RET tyrosine kinase signaling pathway, to treat advanced medullary thyroid carcinoma (60). Growth of CCDC6-RET-positive LC-2 lung adenocarcinoma cells can be inhibited by vandetinib *in vitro* (61). The combination therapy of vandetanib and everolimus can modify efflux mediated by P-gp/Abcb1- and Bcrp1/Abcg2, improve blood-brain barrier penetration, and increase the survival time of patients with brain metastases (62). It also can suppress the transplantation of CCDC6-RET-positive lung adenocarcinoma tumors into athymic mice and the carcinogenesis of KIF5B-RET transgenic mice *in vivo* (63).

Based on existing Phase I and Phase II trials, vandetanib was well tolerated at a single daily dose of 300 mg. Lee et al. (64) conducted a phase II clinical trial in which 18 patients with RET fusion-positive metastatic or recurrent NSCLC who previously received and responded to platinum-based doublet chemotherapy were recruited between July 2013 and October 2015. These patients were given vandetanib 300 mg/day. They had an objective response rate (ORR) of 18%, a median progression-free survival (mPFS) of 4.5 months, and median overall survival (mOS) of 11.6 months at the end of the trial. The most common grade three or higher treatment-related adverse events (TRAEs) were hypertension (89%), rash (72%), diarrhea (44%), acne (28%), and asymptomatic QT prolongation (11%).

Yoh et al. (65) published the final follow-up data of Phase II clinical trial they completed between April 2013 and May 2015 in 19 previously treated RET fusion-positive NSCLC patients in May 2021. These patients had an ORR of 53% (95% CI: 31-74), an mPFS of 6.5 months, and an mOS of 13.5 months. Hypertension (84.2%), diarrhea (78.9%), acneiform rash (63.2%), asymptomatic QT prolongation (47.4%), dry skin (42.1%) were the most prevalent grade three or higher TRAEs. It can be noted that vandetanib has a restricted objective remission rate, no evident advantages in terms of efficacy, and a significant prevalence of grade three or higher TRAEs, which is a fundamental reason for limiting its clinical utilization.

Cabozantinib (XL184) is a multikinase inhibitor, which inhibits RET, VEGFR-1/2/3, MET, ROS1, AXL, and other kinases (66, 67). Cabozantinib has been approved in some locations for RET fusion-positive NSCLC, medullary thyroid cancer and Advanced Renal Cell Carcinoma (68). Nokihara et al. completed a phase I research (69), in which 43 NSCLC patients received cabozantinib 60 mg/day, eight patients achieved varied degrees of remission, and 16 patients experienced stable illness. Hypertension, proteinuria, and venous thrombosis were commonly observed TRAEs.

In June 2013, cabozantinib was used in a phase II study (70) involving three RET fusion-positive NSCLC patients. Two of these patients showed marked partial responses (including one with a TRIM33-RET fusion). Another patient with a KIF5B-RET fusion had a roughly 8-month period of stable illness. Hypertension, proteinuria, and tiredness were among the grade three or higher TRAEs. In 2016, Drilon et al. (71) reported their phase II clinical study involving 26 patients with RET fusion-positive NSCLC, cabozantinib 60 mg/day, and the follow-up results of the 25 patients were ORR: 28%, mPFS: 5.5 months, and mOS: 9.9 months, and the primary grade three or higher TRAEs was increased lipase (15%), increased alanine aminotransferase (8%), decreased platelet count (8%), and hypophosphatemia (8%). Cabozantinib has been indicated to have a general efficacy in patients with RET-positive NSCLC. Still, because its safety is tolerable, it can be utilized as a backup alternative when no other targeted medications with superior efficacy are available.

Lenvatinib is a RET, KIT, FGFR1-4, PDGFRα, and VEGFR-1/2/3 kinase inhibitor. It can be combined with everolimus to treat advanced renal cell carcinoma, radioactive iodine-refractory differentiated thyroid cancer, inoperable hepatocellular carcinoma, and advanced endometrial cancer in combination with pembrolizumab (72–75). Taylor et al. (76) conducted a phase Ib/II trial with 21 NSCLC patients (lenvatinib 24 mg/day plus pembrolizumab). After 24 weeks, the 21 patients had an ORR of 33%, a mPFS of 5.9 months, and a median duration of response (mDOR) of 10.9 months. The most common grade three or higher TRAEs were hypertension (20%), tiredness (12%), diarrhea (9%), proteinuria (8%), and elevated lipase (7%). In another phase II clinical research (77), oral lenvatinib 24 mg/day was provided to 25 patients, 13 of whom were KIF5B-RET and 12 of whom were CCDC6-RET. At the end of follow-up, their overall ORR was 16% mPFS was 7.3 months, and mOS was not reached. Twenty-three (92%) patients had TRAEs of grade three or above, and six (24%) patients had to quit due to more extreme TRAEs. The most prevalent grade three or higher TRAEs was hypertension (68%), and the others are nausea (60%), decreased appetite (52%), diarrhea (52%), and proteinuria (48%). These clinical trials revealed that lenvatinib is ineffective, has a significant rate of TRAEs such as hypertension, nausea, and diarrhea, and has evident limitations in clinical use, rendering it inappropriate for widespread clinical use. **Table 3** presents clinical trials of several non-selective inhibitors.

**Table 3:** Non-selective inhibitors in RET+NSCLC patients.

References	Medicine	Phase	Previous treatment	Patients (N)	ORR (%)	mPFS (months)	mOS (months)
Lee et al., 2017 (64),	Vandetanib 300mg/day	II	pretreated	17	18	4.5	11.6
Yoh et al., 2021 (65),	Vandetanib 300mg/day	II	pretreated	19	53 (95%CI, 31-74)	6.5 (95% CI, 3.9-9.3)	13.5 (95% CI, 9.8-28.1)
Drilon et al., 2016 (66),	Cabozantinib 60mg/day	II	pretreated/untreated	25	28 (95%CI, 12-49)	5.5 (95% CI, 3.8-8.4)	9.9 (95% CI, <8.1)
Hida et al., 2019 (77),	Lenvatinib 24 mg/day	II	pretreated	25	16 (95%CI, 4.5-36.1)	7.3 (95% CI, 3.6-10.2)	——

ORR, objective response rate; mPFS, median progression-free survival; mOS, median overall survival.

### Selective RET fusion-positive inhibitors

Selpercatinib (LOXO-292) is a highly selective RET inhibitor involved in ATP competition (58). It suppresses RET fusions (for example, KIF5B-RET and CCDC6-RET) as well as mutations (for example, V804L, V804M, and M918T) (78). Based on Phase I/II Libretto-001 trial findings, selpercatinib was granted regular approval from the FDA to treat adults with locally advanced or metastatic RET fusion-positive NSCLC (11). Aside from its significant inhibitory action on RET, the medication has demonstrated reasonable target specificity, tolerance, and intracranial effectiveness (79).

In Sep 2022, Drilon et al. (80) published the latest results of their registrational LIBRETTO-001 phase I/II trial, a global multicenter clinical trial. This trial included 247 patients with advanced RET fusion-positive NSCLC who had previously used platinum-based chemotherapy and 69 patients who had never been treated. This time the number of patients was more than double what they reported in 2020 (81), which makes their reported drug effects more convincing. All patients were given selpercatinib 160 mg twice daily. By data cut-off, ORR was found to be 61% in 105 platinum-treated patients, with a mDOR of 28.6 months and an mPFS of 24.9 months. Similar observation results were made in the LIBRETTO-001 clinical trials in Japan and China. The study from China (82) included 26 patients with RET fusion-positive NSCLC, 18 of whom had previously received chemotherapy or immunotherapy, and 8 who were treatment-naïve. The ORR was 61.1% in previously treated patients and 87.5% in treatment-naïve patients, with mPFS and mOS not reached at data cut-off. The ORR was 55.3% (95% CI, 38.3-71.4) among 38 patients in the Japanese study (83) who had previously received chemotherapy or immunotherapy. This means that selpercatinib treatment of RET fusion-positive NSCLC may not be significantly different between different populations.

Surprisingly, 106 patients with initial intracranial metastases achieved an ORR of 85% (95% CI, 65-96). And achieved mPFS (95% CI, 13.8-NR) of 19.4 months, which is very optimistic in the survival status of stage IV lung cancer. In addition, In the 22 responders with measurable CNS metastases, the mDOR was 9.4 months (95% CI, 7.4-15.3). This indicates that selpercatinib has good intracranial reactivity, which is beneficial for prolonging the survival time of NSCLC patients with intracranial metastases is significant.

Similar intracranial hyperresponsiveness to selpercatinib was also demonstrated in another study. Subbiah et al. (84) reported the efficacy of selpercatinib 160 mg twice daily for one year in 80 patients with RET fusion-positive NSCLC with brain metastases. Among 22 patients with evaluable intracranial lesions, intracranial ORR at data cut-off was 82%, and 23% of patients achieved complete remission. A total of 80 patients had an intracranial mPFS of 13.7 months, and the mDOR was not reached at the data cutoff. The good safety profile of selpercatinib was also reflected in the trial by Drilon et al. (80). TRAEs occurred in 44% of patients, the most prevalent grade three or higher TRAEs in these patients after treatment were hypertension (19.7%), ALT increased (11.4%), AST increased (8.8%), diarrhea (5.0%), and electrocardiogram QT prolonged (4.8%). It is not difficult to see from these studies that Selpercatinib has a high response rate, good targeting, fewer side effects, and significant intracranial efficacy. Selpercatinib will be a valuable treatment option for these patients with RET fusion-positive NSCLC, especially those with brain metastases. It will be expected that more clinical data are available to verify its efficacy and safety.

Pralsetinib (BLU667) is another potent and selective RET inhibitor with selective activity against gatekeeper mutations (85), which has a central system proven efficacy in a mouse model (8). ARROW (86) is a 13-country, multi-cohort, open-label phase I/II trial. On August 13, 2022, Griesinger et al. updated their latest analysis (87). A total of 281 RET fusion-positive NSCLC patients were involved between March 17, 2017, and November 6, 2020. These patients received a phase II dose of 400 mg of Pralsetinib once daily, and the endpoints were ORR. Pleasantly, they get good therapeutic results. They got an ORR of 72% in treatment-naïve patients and 59% in patients who had previously received platinum-based chemotherapy. The mDOR in chemotherapy patients was 22.3 months, while the mDOR in treatment-naïve patients was not reached. All treatment-naïve patients and 97% of platinum-based chemotherapy patients had tumor volume reductions after treatment, and their respective progression survival was 13.0 months and 16.0 months. In addition, like Selpercatinib, Pralsetinib also has good intracranial efficacy. The intracranial remission rate was 70%(95% CI 35-93) in patients with intracranial metastases after treatment. The mDOR was 10.5 months (95% CI 5.5-12.6 months). Of all 281 patients, 7% (20/281) discontinued due to TRAEs. In 116 treatment-naïve patients, 08 (93%) patients experienced a TRAE, the most common grade three or higher TRAEs were neutropenia (18%), hypertension (10%), serum creatine phosphokinase Elevated (9%) and lymphopenia (9%). In the pre-treated population, the most common grade three or higher TRAEs were neutropenia (22%), anaemia (18%), and hypertension (13%).

According to the findings of these studies, pralsetinib is a medication with good tolerability and safety, a decent ORR and mPFS, and a low occurrence of side events such as neutropenia and hypertension. However, the incidence of Pralsetinib TRAEs was higher than that of Selpercatinib. In terms of rational use, Pralsetinib remains a preferred option for patients with RET fusion-positive NSCLC.

Alectinib is a second-generation highly selective anaplastic lymphoma tyrosine kinase inhibitor with favourable central nervous system activity (88). Having demonstrated great activity in ALK fusion-positive (89), It has been approved as a first-line treatment for metastatic ALK fusion-positive NSCLC after crizotinib treatment (90). Previous studies have shown that Alectinib strongly inhibits RET, FLT3, CHEK2, and LTK but not VEGFR (91). Therefore, it may also be a potentially effective treatment option for RET fusion-positive NSCLC. Takeuchi et al. (92) conducted I/II experiment in Japan. Between September 28, 2016, and January 29, 2018, 25 patients received 450 mg of alectinib twice daily. The final ORR was 4%, mPFS was 3.4%, and mOS was 19 months. An overall disease control rate of 52% was obtained. Grade three or higher TRAEs included diarrhea, pneumonia, elevated serum creatine phosphokinase and bilirubin, hyponatremia, and neutropenia (all at 3.6%).

Ribeiro et al. (93) investigated the efficacy and tolerability of alectinib in four patients with RET fusion-positive NSCLC who received 600 mg twice daily. All patients had previous chemotherapy and TKI therapy. Three cases contained one KIF5B-RET fusion and one CCDC6-RET fusion. Two months after starting medication, one patient’s condition progressed. Two patients had a PFS of four to five months. No grade three or higher TRAEs occurred in these four patients. Another clinical trial (94) enrolled 14 patients with RET fusion-positive NSCLC and achieved an mPFS of 3.7 months (95% CI, 1.8 - 7.3 months). Unfortunately, the researchers stopped early due to the lack of positive efficacy observed in other studies. Although past preclinical studies have shown that alectinib has a significant inhibitory effect on RET fusion genes (10, 95), but from the results of these clinical trials, alectinib is difficult to offer considerable disease control results in patients with advanced and chemotherapy-refractory RET fusion-positive NSCLC. Nevertheless, it has the advantages of low toxicity and side effects and great potential application value worthy of our research. Considering the small number of patients involved in previous clinical trials, maybe we need a larger clinical trial to verify the efficacy and safety of alectinib. **Table 4** presents clinical trials of several selective inhibitors.

**Table 4:** Selective inhibitors in RET+NSCLC patients.

References	Medicine	Phase	Previous treatment	Patients (N)	ORR (%)	mPFS (months)	mDOR (months)
Drilon et al., 2022 (80),	Selpercatinib 160 mg twice daily	I/II	chemotherapy	247	61 (95% CI, 55-67)	24.9 (95% CI, 19.3-NR)	28.6 (95% CI, 20.4-NR)
		II	untreated	69	84 (95% CI, 73-92)	22.0 (95% CI, 13.8-NR)	20.2 (95% CI, 13.0-NR)
Lu et al., 2022 (82),	Selpercatinib 160 mg twice daily	II	chemotherapy/Immunotherapy	18	61.1 (95% CI, 35.7-82.7)	NR	NR
			untreated	8	87.5 (95%CI, 47.3-99.7)	NR	NR
Subbiah et al., 2021 (84),	Selpercatinib 160 mg twice daily	I/II	brain metastases (treated/untreated)	80	82 (95% CI, 65-95)	13.7 (96% CI, 10.9-NR)	NR (95% CI, 9.3-NR)
Griesinger et al., 2022 (87),	Pralsetinib 400 mg/day	I/II	chemotherapy	136	59 (95% CI, 50-67)	16.5 (95% CI, 10.5-24.1)	22.3 (95% CI, 15.1-NR)
		II	untreated	75	72 (95% CI, 60-82)	13.0 (95% CI, 9.1-NR)	NR (95% CI, 9.0-NR)
Takeuchi et al., 2021 (92),	Alectinib 450 mg twice daily	II	untreated	25	4 (95% CI, 0.1-20.4)	3.4 (95% CI, 2.0-5.4)	NR

ORR, objective response rate; mPFS, median progression-free survival; mDOR, median duration of response; NR, not reached.

### Problems and prospects of RET fusion-positive inhibitors

RET fusion-positive inhibitors have brought new changes to the treatment of NSCLC, bringing new hope to patients with advanced or metastatic NSCLC who cannot be operated on. But they also have many problems, the main one is their TRAEs, such as hypertension, rash, diarrhea, neutropenia, liver function damage, etc. And Kalchiem-Dekel et al. found that Chylothorax and Chylous Ascites may appear during treatment with selected RET TKIs (96). Recognizing this side effect can help identify whether a tumor is progressive. These TRAEs will greatly limit the therapeutic dose of inhibitors, and some people even suffer from severe TRAEs and discontinuation. The problem we need to face is how to choose an appropriate drug and dosage according to the patient’s condition, and balance the factors such as treatment effect, TRAEs, and patient’s quality of life. If the toxic and side effects are too strong and the patient cannot get better disease control effects, we should choose other conservative treatments, which will make the patient’s quality of life better.

**Figure 3:**
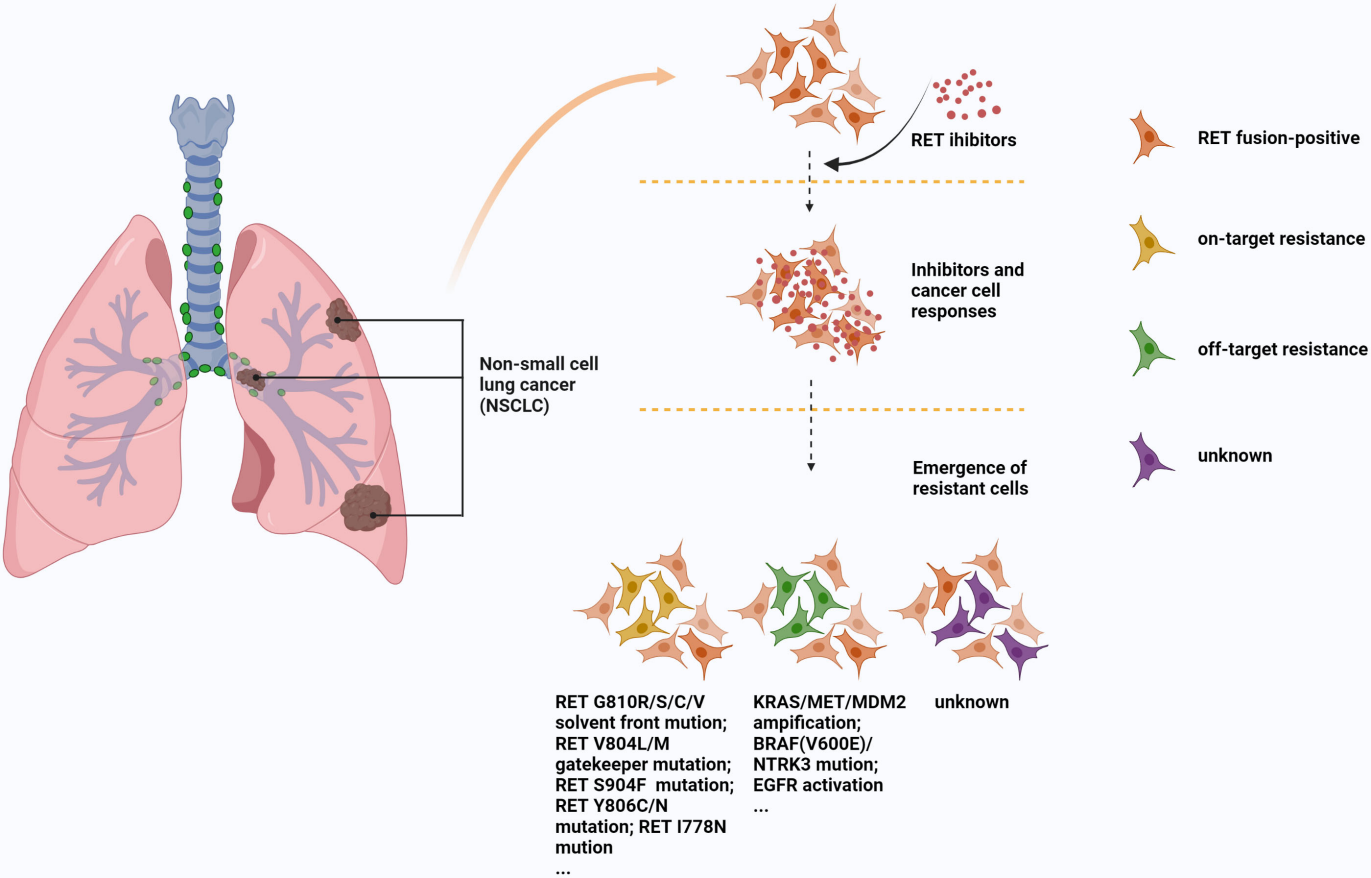
Mechanisms of MKI and TKI resistance in RET fusion-positive NSCLC.

Another important aspect is the problem of resistance to these drugs. The resistance mechanisms of RET inhibitors are mainly composed of on-target mutation, off-target bypass activation or some unknown resistance pathways (97, 98). V804L/M residue gatekeeper mutation of secondary drug resistance mutation is an on-target resistance mechanism usually seen in MKI (97), a study (99) has shown that a novel RET inhibitor, SYHA1815, can overcome this resistance, which may be a new direction for drug development. Some other on-target mechanisms, S904F, G810R/S/C/V residue solvent front mutations, and I788N somatic mutation are also associated with MKI or selective TKI resistance (98, 100–102). Off-target resistance mainly includes KRAS/MET amplification, BRAF(V600E)/NTRK3 mutation, EGFR activation, and MDM2 amplification (78, 103–105), these resistance mechanisms can be seen in MKI or selective TKI. In particular, NTRK3 fusion has achieved post-clinical validation as an acquired resistance mechanism to selpercatinib in RET fusion-positive lung cancer (105). Unlike MKI, selpercatinib and pralsetinib avoid the interference of some gatekeeper mutations (106). However, it is very susceptible to non-gatekeeper mutations (such as RETG810C/S and RETY806C/N mutations). Solomon et al. (100) found 5 resistant patients after using selpercatinib, including RET carrier premutation (G810C/R/S/V) and olvent front trans-gatekeeper mutation. The speed, scope and severity of the mutations are staggering. **Figure 3** shows several common mechanisms by which MKI or selective TKI resistance.

There are some caveats when analyzing TKI resistance. According to Xia, Lin, et al. (107, 108), analyzing tumor re-biopsy acquired resistance pathways is critical for next-generation RET TKIs, especially when there is an acquired resistance mutation spectrum difference between selpercatinib and pralsetinib. Next-generation TKI assays may require discriminating between RET fusion-positive and RET-mutant cancers, and differences in resistance between selpercatinib and pralsetinib. Numerous studies have shown that the combination of different RET fusion-positive inhibitors, or in combination with other drugs, can improve efficacy or overcome some MKI/TKI resistance. Rosen EY et al. (109) discovered that a combination of selpercatinib and crizotinib can overcome MET-dependent resistance in RET fusion-positive NSCLC. Another study (110) also reported that the combination of Pralsetinib and Sequential MET Inhibitors can overcome MET amplification resistance. Cabozantinib combined with everolimus improves the therapeutic effect of advanced renal cell carcinoma (111, 112), which may be a revelation of treatment for patients with RET fusion-positive NSCLC. An important research (113) result in thyroid cancer, inhibition of FGF receptor blocks adaptive resistance to RET inhibition in CCDC6-RET-rearranged thyroid cancer. This may also be replicated in RET fusion-positive NSCLC.

Overall, among non-selective RET fusion-positive inhibitors. Vandetinib and lenvatinib have very low efficacy and are accompanied by relatively serious TRAEs, which do not have much practical application value. Cabozantinib shows some activity against RET fusion-positive, and its safety is tolerable, so it can be used as a substitute or in combination with other drugs when there is no other drug with excellent efficacy. In contrast, selective fusion-positive inhibitors gave us more confidence, especially their good performance in intracranial efficacy. Selecting RET fusion-positive inhibitors not only has good reactivity, but also has relatively milder drug side effects. NCCN guidelines (114) also advocate selpercatinib and pralsetinib as first-line therapies for patients with RET fusion-positive advanced NSCLC. However, the NCCN also pointed out that if RET fusion-positive NSCLC is found, treatment with PD-1/PD-L1 inhibitors should be avoided.

## Conclusions

In summary, we recommend that DNA NGS and RT-PCR should be used as the primary tool for RET fusion detection, but IHC or FISH can be chosen based on economics when screening large samples. RNA NGS can be used to confirm atypical positive and borderline negative FISH results. RNA NGS is the first choice when screening for novel fusion partners, antisense fusions, or intergenic regions is required. For RET fusion-positive inhibitors, the authors emphasize that, based on existing preclinical and clinical evidence, RET fusions have the strong potential to be an important therapeutic target for NSCLC. Selpercatinib and Pralsetinib are now the treatment of choice for patients with RET fusion-positive treatment-naïve and metastatic NSCLC, with cabozantinib available as an alternative or in combination. Alectinib has the potential to help treat RET fusion-positive NSCLC, however, more extensive randomized clinical trials are needed to confirm its efficacy and safety. Besides, it is necessary to further study the resistance mechanism of RET inhibitors and develop a new generation of anti-resistance inhibitors. Exploring the combination of RET inhibitors with other therapies is also recommended for improving overall efficacy and overcoming some resistance.

## Author contributions

(I) Conception and design: ZS, BQ, GL. (II) Administrative support: None. (III) Provision of study materials or patients: None. (IV) Collection and assembly of data: ZS, BQ, LL, BY. (V) Data analysis and interpretation: ZS, BQ, LL, BY, GL. (VI) Manuscript writing: ZS, BQ. (VII) Final approval of manuscript: These authors have contributed equally to this work.
